# PTEN deficiency potentiates HBV-associated liver cancer development through augmented GP73/GOLM1

**DOI:** 10.1186/s12967-024-04976-4

**Published:** 2024-03-08

**Authors:** Fuqiang Huang, Jing Guo, Na Zhao, Mengjie Hou, Xiaochen Gai, Shuhui Yang, Pei Cai, Yanan Wang, Qian Ma, Qi Zhao, Li Li, Huayu Yang, Yanling Jing, Di Jin, Zhongdong Hu, Xiaojun Zha, Hongyang Wang, Yilei Mao, Fangming Liu, Hongbing Zhang

**Affiliations:** 1 State Key Laboratory of Common Mechanism Research for Major Diseases, Haihe Laboratory of Cell Ecosystem, Department of Physiology, Institute of Basic Medical Sciences and School of Basic Medicine, Chinese Academy of Medical Sciences and Peking Union Medical College, 5 Dong Dan San Tiao, Beijing, 100005 China; 2 Department of Blood Transfusion, Shandong Provincial Hospital Affiliated to Shandong First Medical University, Jinan, China; 3 Institute of Cancer Stem Cell, Dalian Medical University, Dalian, China; 4 Modern Research Center for Traditional Chinese Medicine, Beijing Research Institute of Chinese Medicine, Beijing University of Chinese Medicine, Beijing, China; 5 Department of Biochemistry & Molecular Biology, School of Basic Medicine, Anhui Medical University, Hefei, China; 6 Department of Liver Surgery, Peking Union Medical College Hospital, Chinese Academy of Medical Sciences, Beijing, China; 7 International Co-Operation Laboratory On Signal Transduction, Eastern Hepatobiliary Surgery Hospital, Naval Medical University, Shanghai, China

**Keywords:** Hepatocellular carcinoma, Intrahepatic cholangiocarcinoma, Hepatitis B virus, Pten, GP73

## Abstract

**Background:**

Although hepatitis B virus (HBV) infection is a major risk factor for hepatic cancer, the majority of HBV carriers do not develop this lethal disease. Additional molecular alterations are thus implicated in the process of liver tumorigenesis. Since phosphatase and tensin homolog (PTEN) is decreased in approximately half of liver cancers, we investigated the significance of PTEN deficiency in HBV-related hepatocarcinogenesis.

**Methods:**

HBV-positive human liver cancer tissues were checked for PTEN expression. Transgenic *HBV*, *Alb-Cre* and *Pten*^*fl/fl*^ mice were inter-crossed to generate WT, *HBV, Pten*^*−/−*^ and *HBV; Pten*^*−/−*^ mice. Immunoblotting, histological analysis and qRT-PCR were used to study these livers. *Gp73*^*−/−*^ mice were then mated with *HBV; Pten*^*−/−*^ mice to illustrate the role of hepatic tumor biomarker golgi membrane protein 73 (GP73)/ golgi membrane protein 1 (GOLM1) in hepatic oncogenesis.

**Results:**

*Pten* deletion and *HBV* transgene synergistically aggravated liver injury, inflammation, fibrosis and development of mixed hepatocellular carcinoma (HCC) and intrahepatic cholangiocarcinoma (ICC). GP73 was augmented in *HBV; Pten*^*−/−*^ livers. Knockout of GP73 blunted the synergistic effect of deficient *Pten* and transgenic *HBV* on liver injury, inflammation, fibrosis and cancer development.

**Conclusions:**

This mixed HCC-ICC mouse model mimics liver cancer patients harboring HBV infection and PTEN/AKT signaling pathway alteration. Targeting GP73 is a promising therapeutic strategy for cancer patients with HBV infection and PTEN alteration.

**Supplementary Information:**

The online version contains supplementary material available at 10.1186/s12967-024-04976-4.

## Background

Liver cancer is the third leading cause of cancer death worldwide [1, 2]. It comprises hepatocellular carcinoma (HCC), intrahepatic cholangiocarcinoma (ICC), mixed HCC-ICC and other neoplasms [3, 4]. HCC is the dominant primary liver cancer type, accounting for 90% of all cases. ICC is the second common malignancy in liver, with an incidence of approximately 6% of all primary hepatic tumors. It has poorer prognosis than HCC. Hepatobiliary cancer is an infrequent subtype of primary liver cancers, which exhibits much worse prognosis than either HCC or ICC [5, 6]. Once diagnosed, the treatment is limited and the outcome is poor [7]. Since its pathological mechanism is largely unknown, clinically relevant animal models are in need to mimic the initiation and progression of mixed HCC-ICC.

Viral infection is a major risk factor for primary liver cancer. Following chronic infection of hepatitis B virus (HBV) or hepatitis C virus (HCV), hepatocytes experience injury, inflammation, fibrosis, cirrhosis, and eventually carcinogenesis [6–8]. In the United States, Europe, and Japan, most of liver cancers are associated with HCV infection. Due to excellent virological response with antiviral drugs, the risk of developing HCV infection-associated liver cancer has declined substantially. In contrast, HBV infection is still the most prominent risk factor for HCC development [7]. In China, some regions of Asia and Africa, HBV-related liver cancer is prevalent [9]. Half of global liver cancer occurs in China because up to 10% of its population are HBV carriers [10–12]. Although liver cancer development is highly correlated with HBV infection, only a small percentage of HBV carriers finally develop liver cancer after a long latency [13, 14]. This suggests that additional molecular alterations are involved in HBV-related liver cancer progression. Therefore, illumination of the essential molecular events that triggered this malignant transformation may help us gain insight into the mechanisms and potential treatment for HBV-associated liver cancer.

More than half of hepatic cancers exhibit reduced expression of phosphatase and tensin homolog (PTEN) [15, 16]. Low expression of PTEN confers poor prognosis of HCC patients [17]. PTEN exercises its tumor suppressor function mainly by antagonizing phosphatidylinositol 3-kinase (PI3K) pathway through dephosphorylation of PIP3 [18]. Loss of PTEN activates mechanistic target of rapamycin (mTOR) through active AKT serine/threonine kinase (AKT)-mediated suppression of TSC1 and 2 complex, a suppressor of mTOR [19, 20]. Liver-specific *Pten* deletion mice develop liver cell adenomas at 11 months of age and HCC by 18.5–19.5 months of age [21]. The long latency of cancer initiation and development implies that additional genetic and/or epigenetic alterations likely take place in the liver in the absence of *Pten*. It remains elusive for the role of *Pten* loss in HBV-associated liver cancer initiation and progression.

Golgi membrane protein 73 (GP73)/Golgi membrane protein 1 (GOLM1) is a type II Golgi transmembrane protein [22]. It is a serum marker for liver cancer. Serum GP73 level increases gradually during the process of hepatic HBV infection, cirrhosis and cancer [23–25]. With subcutaneous tumor formation and tail-vein injection assays, we demonstrate that overexpression of GP73 is critical for xenografting HCC tumorigenesis and metastasis triggered by mTOR activation in immune-deficient nude mice [26]. Using human HCC cell lines in culture and in nude mice, Ye et al. showed that GP73 promoted HCC metastasis by modulating epidermal growth factor receptor (EGFR)/RTK cell-surface recycling. Furthermore, the abundance of GP73 is well correlated with human HCC metastasis and poor survival of HCC patients [27]. Although augmented GP73 is highly correlated with HBV infection and HCC, the involvement of GP73 in HBV-mediated primary liver cancer development in immune-competent mice remains to be documented.

The genome of *HBV* encodes four core proteins, including HBV surface antigen (HBsAg), core antigen (HBcAg), DNA polymerase, and X protein (HBx) [28]. Since mouse does not have receptor for HBV, HBV infection does not occur in mice. *HBV* transgenic mice which contain pre-S, S, and X domains of HBV genome develop primary liver cancer around the age of 12 months [29, 30]. Even though HBV infection is not involved in the disease progression in transgenic mice, severe liver injury and inflammation still simulate the pathogenesis in liver cancer patients with HBV infection. Thus, this mouse model is widely used to illustrate the pathologic and molecular events of HBV-mediated tumorigenesis [31–33]. To simulate liver cancer patients with both HBV infection and activation of PI3K/AKT/mTOR signaling pathway in mice, we disrupted *Pten* in the livers of *HBV* transgenic mice and investigated the role of GP73 in liver tumor development of *HBV*; *Pten*^−/−^ mice.

## Methods

### ***Generation of HBV; Pten***^***−/−***^*** and HBV; Pten***^***−/−***^***; GP73***^***−/−***^*** mice***

*HBV* mice (stock no. 002226) were described previously [29]. *Alb-Cre* (stock no. 003574), *Pten*^*fl/fl*^ (stock no. 006068) and *EIIA*-*Cre* (stock no. 003724) mice were from Jackson Laboratory (Bar Harbor, ME, USA). *HBV* and *Alb-Cre* mice were respectively crossed with *Pten*^*fl/fl*^ mice to generate *HBV; Pten*^*fl/fl*^ and *Alb-Cre; Pten*^*fl/fl*^ mice. By intercrossing *HBV; Pten*^*fl/fl*^ mice with *Alb-Cre; Pten*^*fl/fl*^ mice, *HBV; Alb-Cre; Pten*^*fl/fl*^ (*HBV; Pten*^*−/−*^), *Alb-Cre; Pten*^*fl/fl*^ (*Pten*^*−/−*^), *HBV; Pten*^*fl/fl*^ (*HBV*) and *Pten*^*fl/fl*^ (WT) mice were generated. *GP73*^*fl/*+^ mice were developed by Beijing Biocytogen (China) and were mated with *EIIA*-*Cre* mice to generate global GP73 knockout (*GP73*^*−/−*^) mice. To obtain *HBV; Pten*^*−/−*^*; GP73*^*−/−*^ mice, *HBV; Pten*^*−/−*^ mice were crossed with *GP73*^*−/−*^ mice. All mice were in C57BL/6 background and maintained in a pathogen-free facility under a 12-h light/dark cycle with appropriate temperature and humidity. The genotypes of mice were determined by PCR analysis of tail genomic DNA. The primers were shown in Table 1. The animal experiments were approved by Animal Care and Use Committee of Peking Union Medical College and performed in accordance with international guidelines. All of the mice used for this study are male unless as specified.

**Table 1:** Genotyping primer sequences

Primer name	Sequences
*Cre*-F	TGGGCGGCATGGTGCAAGTT
*Cre*-R	CGGTGCTAACCAGCGTTTTC
*HBV*-1	AACATGGAGAACATCACATC
*HBV*-2	AGCGATAACCAGGACAAGTT
*HBV*-3	ATGTACTGGTCCCGCATGGC
*HBV*-4	TTTGCAGGACTCCTACCGG

### Human liver cancer specimens

Human liver cancer tissues and adjacent normal liver tissues were obtained from the patients undergoing resection at Peking Union Medical College Hospital. Written informed contents were obtained from all patients. All of these patients were HBV positive.

### Histological analysis

Mouse liver tissues were fixed in 4% formalin. 5 μm of paraffin-embedded sections of the same liver lobes from different mice were subjected to hematoxylin–eosin (H&E) staining through the following procedures: 1. Dewaxing and hydration with environmental friendly Dewaxing Transparent Liquid, Anhydrous ethanol, 75% Ethyl alcohol and tap water; 2. Fixing with tissue fixation solution; 3. Hematoxylin staining; 4. Eosin staining; 5. Dehydration and sealing. Liver fibrosis was determined by Masson’s trichrome staining kit (Sigma-Aldrich, HT15, St Louis, USA), according to the manufacturers’ protocols. For immune-histochemical analysis, the paraffin sections were subjected to dewaxing to water, antigen repair, endogenous peroxidase blocking, serum closure, primary antibody and secondary antibody incubation, DAB color development and nuclus restaining. Antibody information was listed as follow: CK19 (1:500 dilution, Servicebio, 11197, Wuhan, China), Heppar-1 (1:500 dilution, ZSGB-BIO, 0131, Shanghai, China), CD34 (1:500 dilution, Abcam, 81289, Cambridge, UK), and Cleaved-caspase 3 (1:200 dilution, Cell Signaling Technology, #9664, Danvers, USA). Quantification of fibrotic areas and microvessel areas was determined by Image J software.

### Immunoblotting

Immunoblotting of liver tissues was processed according to the protocols as described previously [26]. Liver tissues were prepared in lysis buffer (2% SDS, 10% glycerol, 10 mM Tris (pH 6.8), and 100 mM DTT) containing Protease and Phosphatase Inhibitor Cocktail (#P002, New cell & molecular, Suzhou, China). Equal amounts of total proteins were separated on 8–12% SDS-PAGE gels, followed by immunoblotting onto the PVDF membranes. Membranes were first incubated with primary antibodies overnight at 4 °C, then incubated with secondary antibodies for 2 h at room temperature. Primary antibodies against PTEN (#9559), pAKT (#4060), AKT (#4691), cleaved-caspase 3 (#9664), JNK (#9252), pSTAT3 (#9131), STAT3 (#9137) were purchased from Cell Signaling Technology, pJNK (131499) was from Abcam, mouse GP73 (customized), GAPDH (AC001) were from Abclonal (Wuhan, China), and human GP73 (66331-1-Ig) was from Proteintech (Rosemont, USA). The detection of protein bands was carried out using LI-COR Odyssey Infrared Scanner.

### RNA preparation and quantitative Real-Time polymerase chain reaction (qRT-PCR)

Total RNA was extracted from mouse liver tissues using Trizol (Invitrogen, Waltham, USA) and Ultrapure RNA kit (CWBIO, Taizhou, China), according to the manufacturers’ protocols. 1 μg of total RNA was reverse-transcribed using ReverTra Ace® qPCR RT Master Mix with gDNA Remover (TOYOBO, FSQ-301, Osaka, Japan). qRT-PCR was performed on a Bio-Rad IQ5 machine with SYBR Green PCR Master Mix (TransGen Biotech, AQ141, Beijing, China). The procedures of qPCR assay were as follow: 1. 95 ℃ for 3 min; 2. 95 ℃ for 15 s; 3. 60℃ for 1 min + Plate Read; 4. GOTO 2, 40 cycles; 5. Melt Curve 55–95 ℃, increment 0.5 ℃ For 0:10 + Plate Read. GAPDH served as internal control. The primer sequences were listed in Table 2.

**Table 2:** qPCR primer sequences

Gene name	Forward (5′-3′)	Reverse (5′-3′)
*Tgfb1*	CTTCCCGAATGTCTGACGTA	GACCGCAACAACGCCATCT
*Ctgf*	TGACCTGGAGGAAAACATTAAGA	AGCCCTGTATGTCTTCACACTG
*Il6*	TTCCATCCAGTTGCCTTCTTGG	TTCTCATTTCCACGATTTCCCAG
*Gapdh*	CATGGAGAAGGCTGGGGCTC	AACGGATACATTGGGGGTAG

### Biochemical analysis

Blood of mice was collected and centrifuged at 3000 rpm for 5 min. Supernatants were collected and sent to the clinical laboratory at Peking Union Medical College Hospital for alanine aminotransferase (ALT) and aspartate aminotransferase (AST) measurement.

### Statistical analysis

Relative mRNA expression, tumor free, mouse survival, tumor burden and histological results were analyzed by GraphPad Prism 9.0 software. Data were shown as mean ± SD. Tumor free or survival was compared using a log-rank (Mantel-Cox) test. Statistical significance between two groups was subjected to Student’s two-tailed* t* tests. One-way ANOVA was used to determine the difference more than two groups. *p < *0.05* was considered statistically significant.

## Results

### PTEN expression is reduced in HBV-related liver cancer

**Fig. 1:**
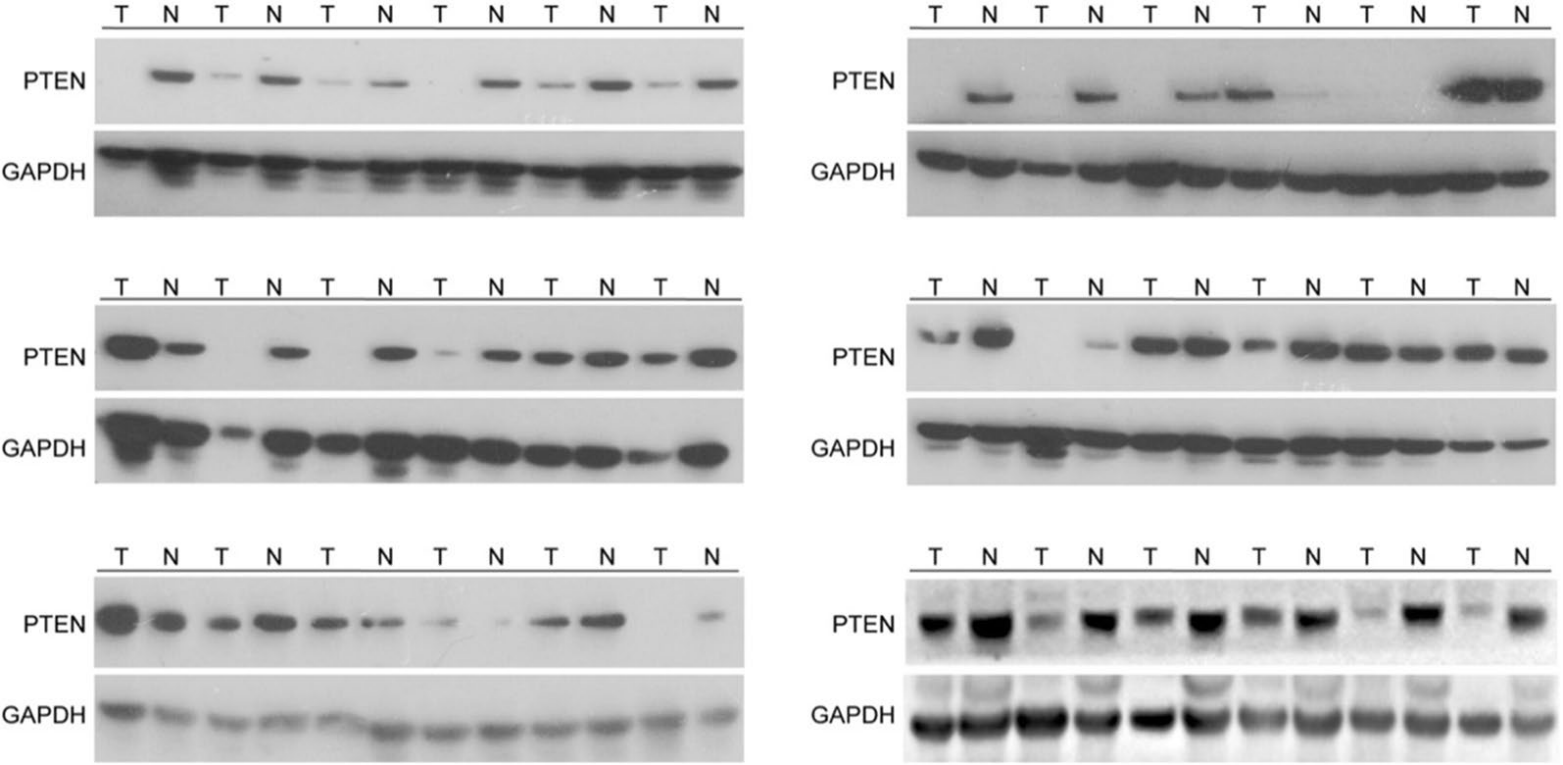
Reduction of PTEN expression in human liver cancer tissues. Immunoblotting analysis of human liver cancer and adjacent tissues. *T* tumor, *N* adjacent tissues

To explore the relevance of PTEN in HBV-related liver cancer, the expression of PTEN was checked in 36 pairs of tumors and adjacent tissues of HBV-positive HCC patients. Comparing with adjacent tissues, PTEN expression was decreased in 72.2% (26/36) cancer samples (Fig. 1).

### Knocking out Pten accelerates HBV-induced liver cancer development

**Fig. 2:**
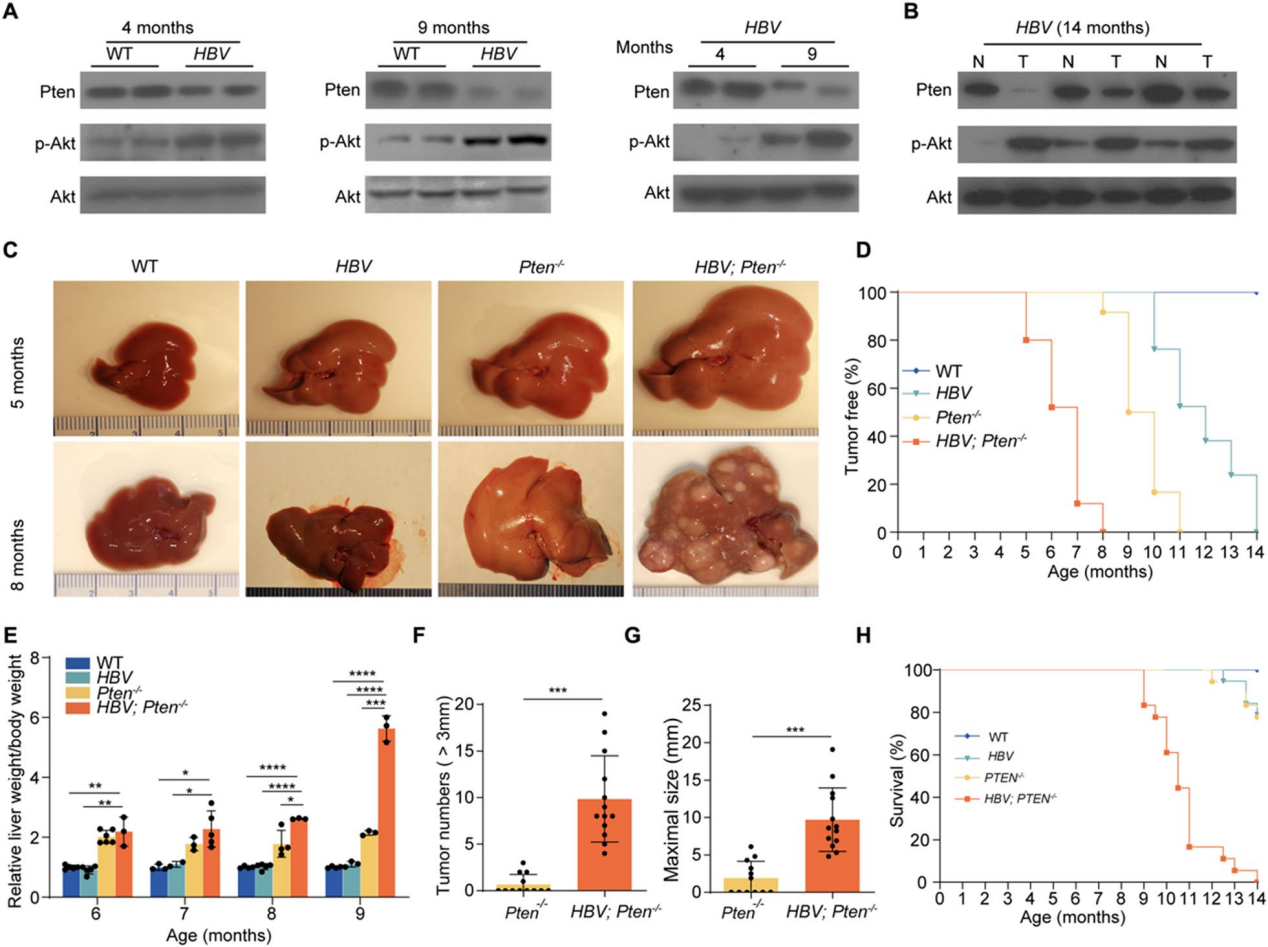
*Pten* deficiency promotes liver tumor formation of *HBV* transgenic mice. **A** Immunoblotting analysis of the livers of WT mice and *HBV* transgenic mice. **B** Immunoblotting analysis of liver tumors and their adjacent liver tissues in *HBV* transgenic mice. T: tumor, N: adjacent non-tumor tissues. **C** Representative images of livers from WT*, HBV, Pten*^*−/−*^*,* and *HBV; Pten*^*−/−*^ mice. **D** Tumor free of WT*, HBV, Pten*^*−/−*^*,* and *HBV; Pten*^*−/−*^ mice (n = 21–25). **E** Ratio of liver weight to body weight for WT*, HBV, Pten*^*−/−*^*,* and *HBV; Pten*^*−/−*^ mice with various ages (n = 3–6). **F** Number (> 3 mm) and **G** maximal size of liver tumors in 9-month-old *Pten*^*−/−*^ (n = 12)*,* and *HBV; Pten*^*−/−*^ (n = 13) mice. **H** Survival of WT*, HBV, Pten*^*−/−*^*,* and *HBV; Pten*.^*−/−*^ mice (n = 18–20). Data were shown as mean ± SD, *p < *0.05*, **p < *0.01*, ***p < *0.001*

We next explored the involvement of PTEN in the progression of HBV-related HCC. Initially, we noticed reduced PTEN and increased pAKT in 4-month-old *HBV* transgenic mouse livers comparing with wildtype (WT) mouse livers. Suppressed PTEN expression and increased AKT phosphorylation were more prominent in 9-month-old *HBV* livers (Fig. 2A). Furthermore, PTEN decreased and pAKT increased in 14-month-old *HBV* transgenic mouse liver tumor compared to adjacent tissues (Fig. 2B). We thus investigated the role of PTEN in HBV-mediated hepatocarcinogenesis. *Pten*^*fl/fl*^ mice were crossed with *HBV* and *Alb-Cre* mice to generate *HBV; Pten*^*fl/fl*^ and *Alb-Cre; Pten*^*fl/fl*^ mice. After mating *HBV; Pten*^*fl/fl*^ mice with *Alb-Cre; Pten*^*fl/fl*^ mice, mice with 4 genotypes were generated, including *Pten*^*fl/fl*^ (WT), *HBV; Pten*^*fl/fl*^ (*HBV*), *Alb-Cre*; *Pten*^*fl/fl*^ (*Pten*^*−/−*^), and *HBV; Alb-Cre; Pten*^*fl/fl*^ (*HBV; Pten*^*−/−*^) mice (Additional file 1: Fig. S1). At age of 5 months, neither *Pten*^*−/−*^ nor *HBV* mice developed visible liver tumors while small lumps were observed in *HBV; Pten*^*−/−*^ mouse livers (Fig. 2C). At 8 months age, all (25/25) of *HBV; Pten*^*−/−*^ mice presented multiple macroscopic tumor foci, whereas none of *HBV* mice and only 8.3% (2/24) *Pten*^*−/−*^ mice exhibited visible tumors (Fig. 2C, D). All of (24/24) *Pten*^*−/−*^ or (21/21) *HBV* mice developed tumors by 11 months or 14 months, respectively (Fig. 2D). Comparing with WT, *HBV*, and *Pten*^*−/−*^ mice, the liver weight to body weight ratio was highest in *HBV; Pten*^*−/−*^ mice at all indicated time points (*p* < *0.05*, Fig. 2E). A total of 9.85 ± 4.63 tumors were presented in 9-month-old *HBV; Pten*^*−/−*^ mice, while only 0.67 ± 1.07 were formed tumors in *Pten*^*−/−*^ mice (*p* < *0.001*, Fig. 2F). The maximal tumor sizes were 9.71 ± 4.25 mm in 9-month-old *HBV; Pten*^*−/−*^ mice and 1.90 ± 2.25 mm in *Pten*^*−/−*^ mice (*p* < *0.001*, Fig. 2G). *HBV; Pten*^*−/−*^ mice began to die at the age of 9 months and none of these mice survived beyond 14 months. Only 22% (4/18) of *Pten*^*−/−*^ mice and 21% (4/19) of *HBV* mice died up to 14 months (Fig. 2H). Taken together, *Pten* deficiency accelerates the initiation and progression of HBV-induced liver cancers and confers these tumor-bearing mice with poor prognosis.

### ***Enhanced fibrosis, micro-vessel formation and combined HCC-ICC in HBV; Pten***^***−/−***^*** mouse livers***

**Fig. 3:**
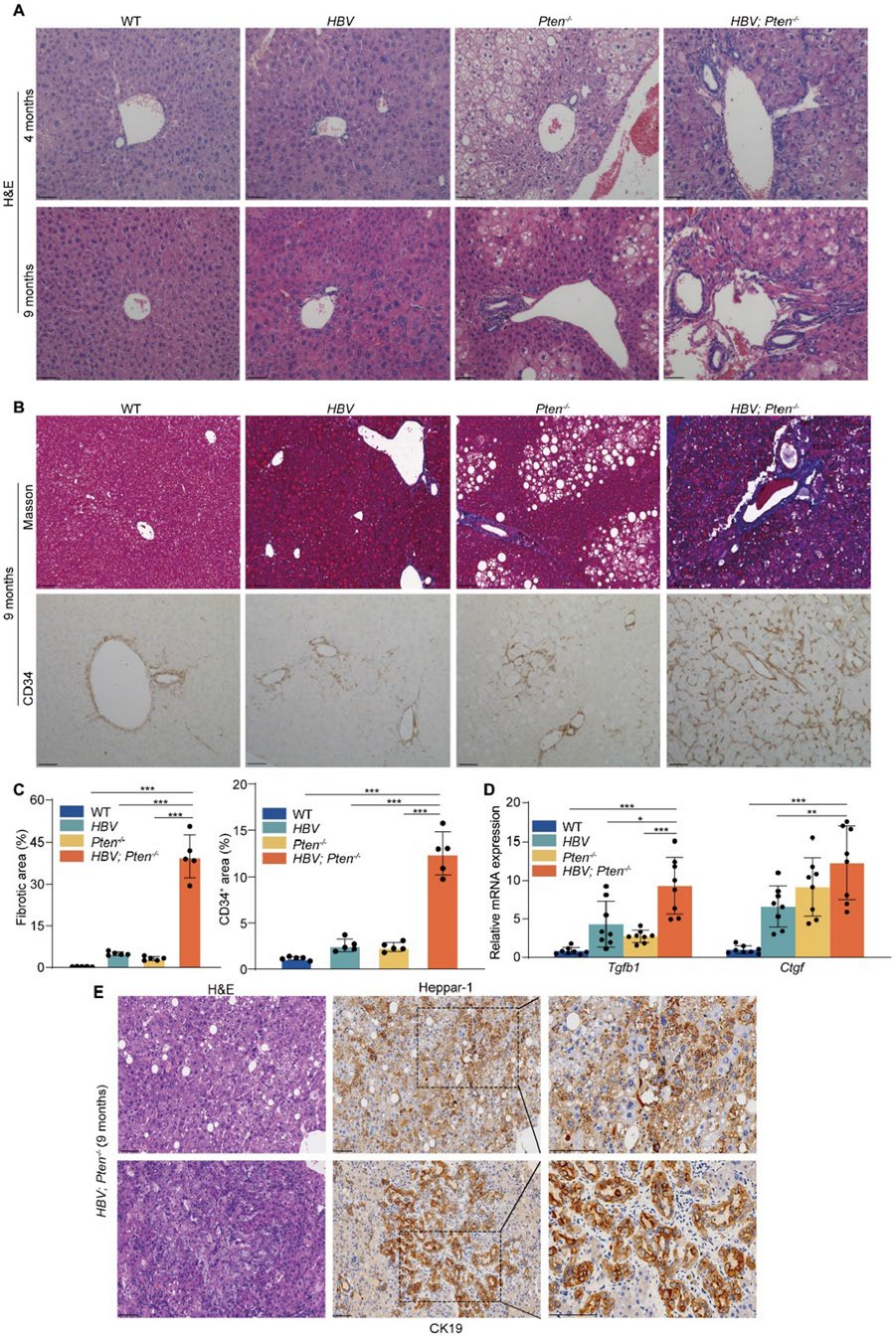
Tumors in *HBV; Pten*^*−/−*^ mice are mixed HCC-ICC. **A** H&E staining of livers from WT*, HBV, Pten*^*−/−*^ and *HBV; Pten*^*−/−*^ mice. **B** Masson’s trichrome and CD34 staining of 9-month-old WT*, HBV, Pten*^*−/−*^*,* and *HBV; Pten*^*−/−*^ mouse livers. **C** Quantitation of Masson’s trichrome and CD34-positive area (n = 5). **D** *Tgfb1* and *Ctgf* mRNA expression in 9-month-old WT*, HBV, Pten*^*−/−*^*,* and *HBV; Pten*^*−/−*^ livers (n = 8–10). **E** H&E, Heppar-1 and CK19 staining of 9-month-old *HBV; Pten*.^*−/−*^ livers. Scale bar, 100 μm. Data were shown as mean ± SD, *p < *0.05*, **p < *0.01*, ***p < *0.001*

To document liver cancer progression in mice, we performed histological analysis of WT, *HBV*, *Pten*^*−/−*^, and *HBV; Pten*^*−/−*^ livers at different stages. Four-month-old *HBV; Pten*^*−/−*^ livers were presented with characteristics of bile duct hyperplasia and inflammatory cell infiltration. The pathological difference between *HBV; Pten*^*−/−*^ livers and other genotypic livers became wider at the age of 9 months (Fig. 3A). Masson and CD34 staining showed increased liver fibrosis and micro-vessel in *HBV; Pten*^*−/−*^ livers (*p* < *0.001*, Fig. 3B, C). Comparing with other three genotypic livers, *HBV; Pten*^*−/−*^ livers had higher expression of fibrosis-associated genes *Tgfb1* and *Ctgf* (*p* < *0.05*, Fig. 3D). HCCs and ICCs were more abundant in 9-month-old *HBV; Pten*^*−/−*^ mice as tumor samples were positive for hepatocyte paraffin 1 (Heppar-1) (HCC biomarker) and CK19 (ICC biomarker) (Fig. 3E) [34]. Collectively, *Pten* deletion accelerates the initiation and progression of HCC and ICC in *HBV* mice.

### ***HBV; Pten***^***−/−***^*** mice exhibit enhanced hepatocyte injury, apoptosis and inflammation***

**Fig. 4:**
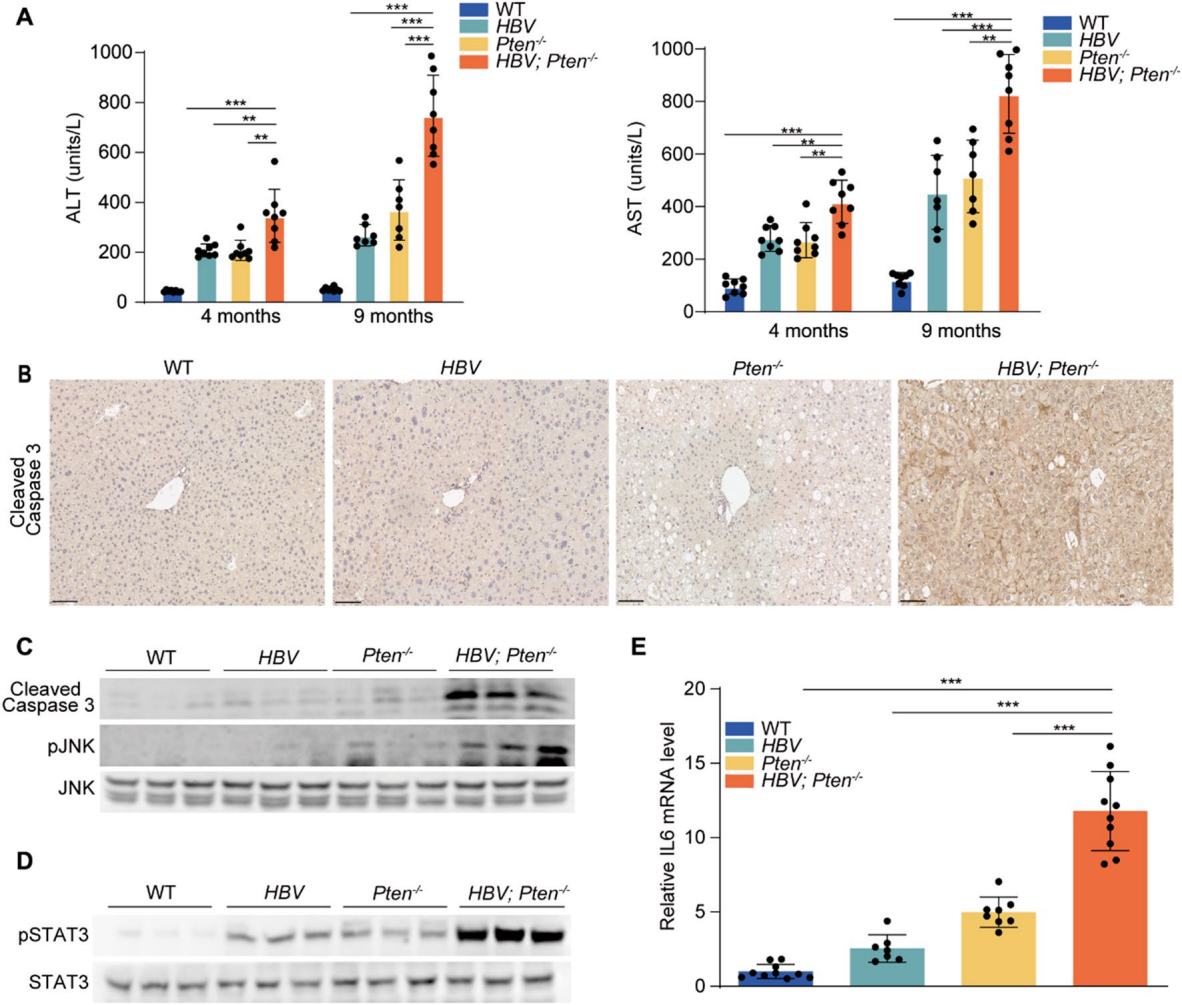
Increased hepatocyte injury, apoptosis and inflammation in *HBV; Pten*^*−/−*^ livers. **A** Serum ALT and AST of WT*, HBV, Pten*^*−/−*^*,* and *HBV; Pten*^*−/−*^ mice. **B** Cleaved-caspase 3 staining of 4-month-old WT*, HBV, Pten*^*−/−*^*,* and *HBV; Pten*^*−/−*^ livers. **C** and **D** Immunoblotting analysis of 4-month-old WT*, HBV, Pten*^*−/−*^*,* and *HBV; Pten*.^*−/−*^ livers. **E** IL6 mRNA level was determined by qRT-PCR (n = 7–10). Scale bar, 100 μm. Data were shown as mean ± SD, **p < *0.01*, ***p < *0.001*

To assess liver injury, ALT and AST were analyzed in the serum of mice with 4 genotypes at the ages of 4 and 9 months. ALT and AST were higher in *HBV* or *Pten*^*−/−*^ mice than in WT mice (*p* < *0.01*, Fig. 4A). The highest levels of ALT and AST were observed in *HBV; Pten*^*−/−*^ mice (*p* < *0.01*, Fig. 4A). Augmented Cleaved-caspase 3 and pJNK in 4-month-old *HBV; Pten*^*−/−*^ mouse livers indicated increased apoptosis (*p* < *0.01*, Fig. 4B, C). Additionally, elevated signal transducer and activator of transcription 3 (STAT3) activity and interleukin 6 (IL6) level indicated severer inflammation in *HBV; Pten*^*−/−*^ mouse livers (*p* < *0.001*, Fig. 4D, E). Collectively, *Pten* deficiency boosts liver injury, hepatocyte apoptosis and inflammation in *HBV* transgenic mice.

### ***Removal of GP73 suppresses liver injury, inflammation, and tumor development in HBV; Pten***^***−/−***^*** mice***

**Fig. 5:**
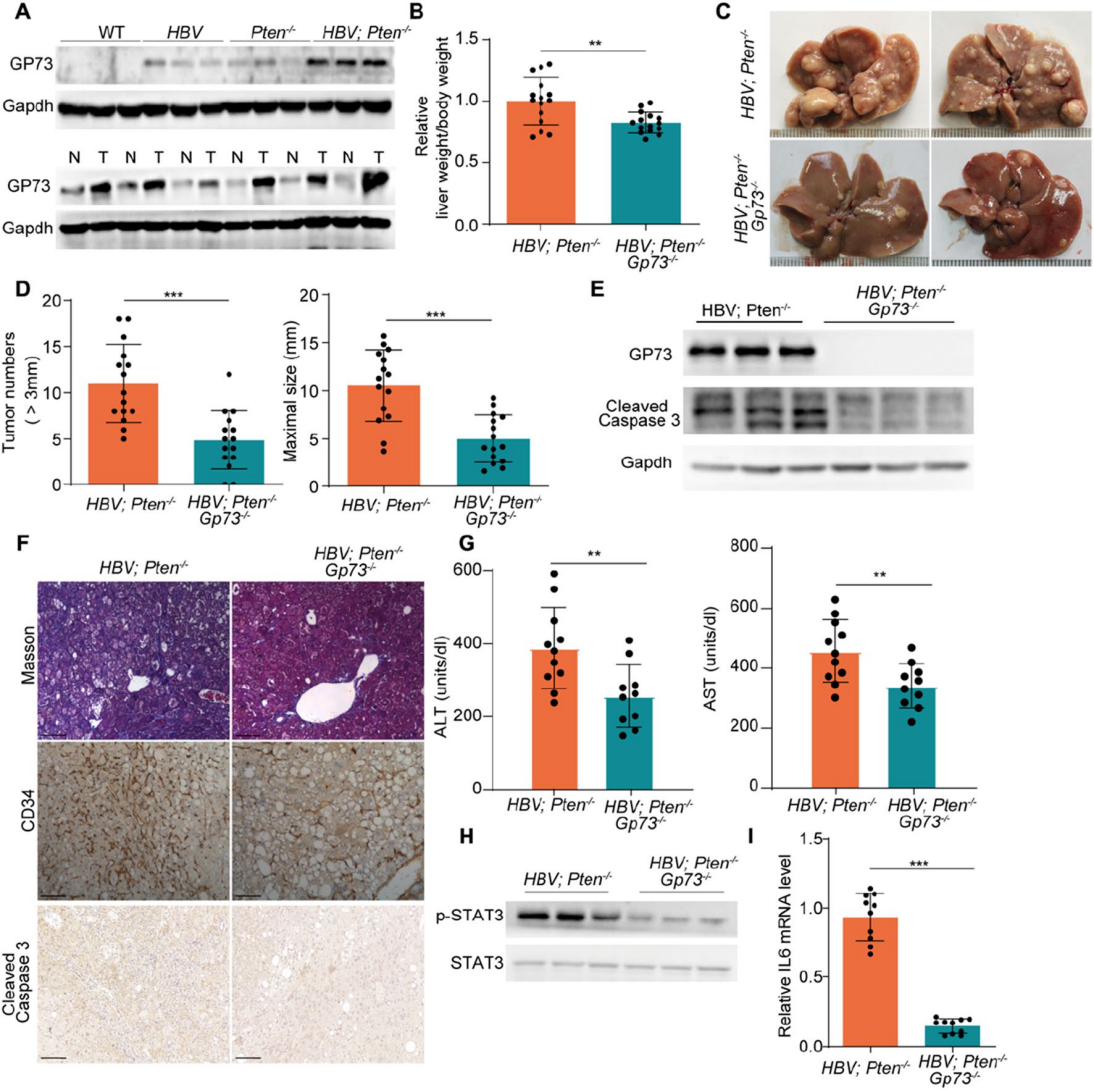
GP73 knockout blocks tumor development of *HBV; Pten*^*−/−*^ mice. **A** Immunoblotting analysis of 4-month-old WT, *HBV*, *Pten*^*−/−*^, and *HBV; Pten*^*−/−*^ livers (upper panel) and tumors and adjacent liver tissues from 9-month-old *HBV; Pten*^*−/−*^ mice (lower panel). T: tumor, N: adjacent tissues. **B**–**D** 9-month-old *HBV; Pten*^*−/−*^ and *HBV; Pten*^*−/−*^*; Gp73*^*−/−*^ mice. **B** The ratio of liver weight to body weight of mice (n = 15). **C** Representative images of livers. **D** Number (> 3 mm) and maximal size of liver tumors (n = 15). **E** Immunoblotting analysis of GP73 and cleaved-caspase 3 of 4-month-old *HBV; Pten*^*−/−*^ and *HBV; Pten*^*−/−*^*; Gp73*^*−/−*^ livers. **F** Masson’s trichrome, CD34 and Cleaved Caspase 3 staining of 9-month-old *HBV; Pten*^*−/−*^ and *HBV; Pten*^*−/−*^*; Gp73*^*−/−*^ livers. **G** Serum ALT and AST of 4-month-old *HBV; Pten*^*−/−*^ and *HBV; Pten*^*−/−*^*; Gp73*^*−/−*^ mice (n = 10–11). **H** Immunoblotting analysis of 4-month-old *HBV; Pten*^*−/−*^ and *HBV; Pten*^*−/−*^*; Gp73*^*−/−*^ livers. **I** qRT-PCR analysis of IL6 mRNA level in 4-month-old *HBV; Pten*^*−/−*^ and *HBV; Pten*^*−/−*^*; Gp73*.^*−/−*^ livers (n = 10). Scale bar, 100 μm. Data were shown as mean ± SD, **p < *0.01*, ***p < *0.001*

To explore the involvement of GP73 in the HCC progression in *HBV; Pten*^*−/−*^ mice, we checked the protein expression of GP73 in the mouse livers with different genotypes. GP73 abundance was slightly elevated in *HBV* or *Pten*^*−/−*^ livers and dramatically increased in *HBV; Pten*^*−/−*^ livers (Fig. 5A, upper panel). GP73 level was further increased in liver tumors relative to adjacent tissues of 9-month-old *HBV; Pten*^*−/−*^ mice (Fig. 5A, lower panel).

To determine whether overexpressed GP73 contributes to accelerated tumor development in *HBV; Pten*^*−/−*^ mice, global GP73 knockout (*Gp73*^*−/−*^) mice were crossed with *HBV; Pten*^*−/−*^ mice to generate *HBV; Pten*^*−/−*^ and *HBV; Pten*^*−/−*^; *Gp73*^*−/−*^ mice. The liver to body weight ratio of *HBV; Pten*^*−/−*^; *Gp73*^*−/−*^ mice was 20% lower than that of *HBV; Pten*^*−/−*^ mice (*p* < *0.01*, Fig. 5B). Furthermore, GP73 knockout profoundly reduced the tumor number (*HBV; Pten*^*−/−*^ vs *HBV; Pten*^*−/−*^; *Gp73*^*−/−*^: 11 ± 4.23 vs 4.87 ± 3.18) and size (*HBV; Pten*^*−/−*^ vs *HBV; Pten*^*−/−*^; *Gp73*^*−/−*^: 10.54 ± 3.73 vs 4.98 ± 2.50) of *HBV; Pten*^*−/−*^ mice (*p* < *0.01*, Fig. 5C, D). Thus, knockout of GP73 prevents hepatocarcinogenesis of *HBV; Pten*^*−/−*^ mice.

To further analyze the role of GP73 in liver tumorigenesis, we examined the molecular and pathologic changes imposed by GP73 knockout in the livers of *HBV; Pten*^*−/−*^ mice. Comparing with *HBV; Pten*^*−/−*^ livers, decreased cleaved-caspase 3 indicated reduced cell apoptosis in *HBV; Pten*^*−/−*^; *Gp73*^*−/−*^ livers (Fig. 5E, F). GP73 knockout also alleviated fibrosis and micro-vessel formation in *HBV; Pten*^*−/−*^ livers (Fig. 5F). In addition, liver function was improved in *HBV; Pten*^*−/−*^; *Gp73*^*−/−*^ mice (*p* < *0.01*, Fig. 5G). The enhanced STAT3 phosphorylation and augmented IL6 in *HBV; Pten*^*−/−*^ livers were reversed by GP73 knockout (*p* < *0.001*, Fig. 5H, I). Our data suggest that GP73 promotes liver cancer through participation of hepatocyte injury, apoptosis and inflammation in *HBV; Pten*^*−/−*^ mice.

### Association between the expression of PTEN and GP73 and the prognosis of HCC patients

**Fig. 6:**
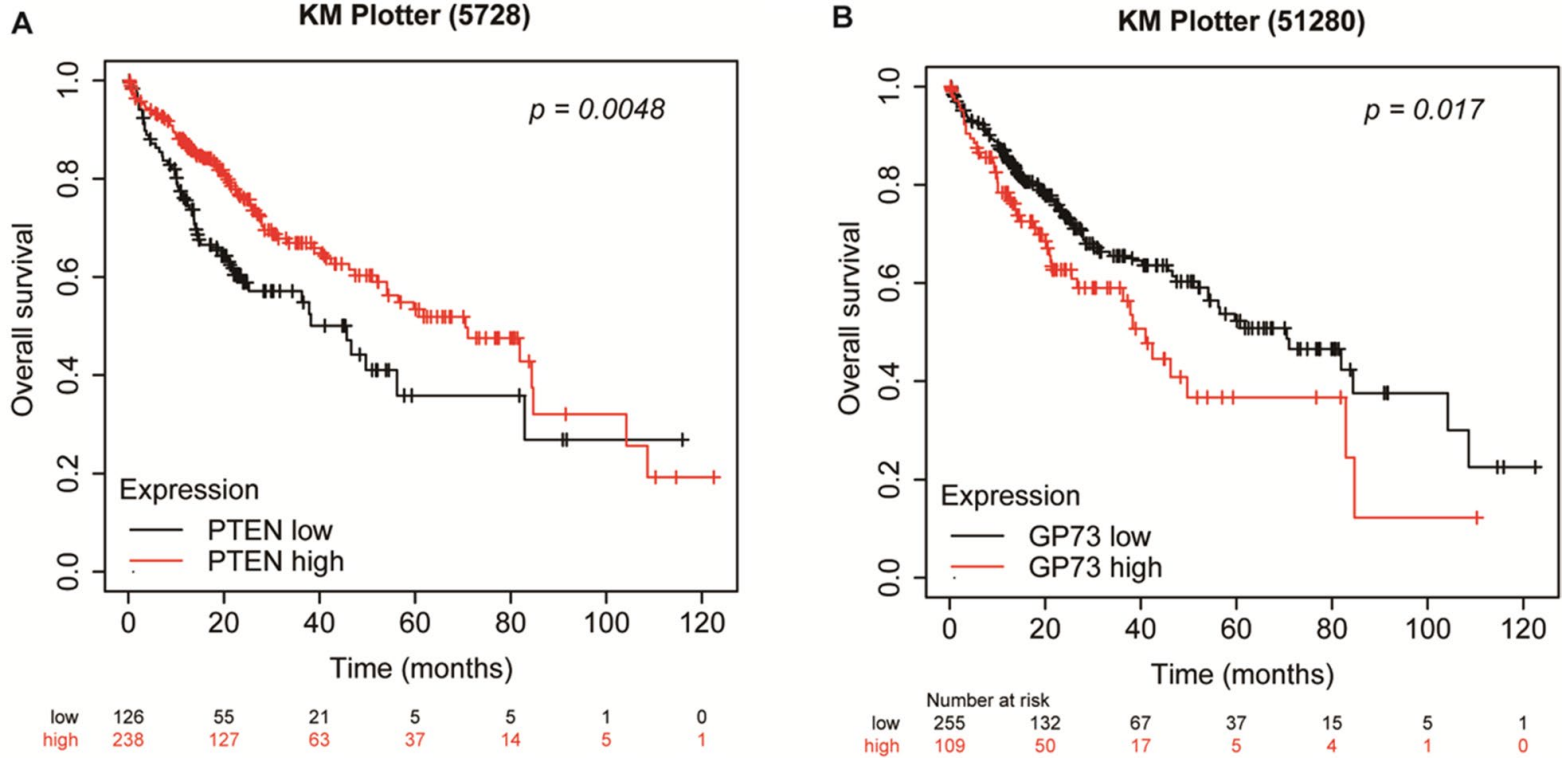
Association between the expression of PTEN and GP73 and the prognosis of HCC patients. **A**–**B** The overall survival of HCC patients with various expression of PTEN **A** (*p* = *0.0048*) or GP73 **B** (*p* = *0.017*) from KM Plotter website

To seek the clinical relevance of PTEN and GP73 in human liver cancer, we analyzed patient survival and the expression of PTEN and GP73 in liver cancer database from KM Plotter website. We found that reduced PTEN and elevated GP73 associated with poor prognosis of HCC patients (Fig. 6).

## Discussion

To investigate the involvement of aberrant PTEN-PI3K/AKT/mTOR signaling cascade in HBV-associated liver cancer, we studied cancer development of liver-specific *HBV* transgenic and *Pten* deletion mice. *HBV* transgenic mice developed tumors between 10 and 14 months while hepatic *Pten* disruption caused tumors between 8 to 11 months. In contrast, *HBV; Pten*^*−/−*^ mice developed mixed HCC-ICC between 5 to 8 months. All *HBV; Pten*^*−/−*^ mice died between 9 to 14 months, whereas 22% (4/18) of *Pten*^*−/−*^ mice died between 12 to 14 months and 21% (4/19) of *HBV* mice died between 12.5 and 14 months. Boosted GP73 was critical for the accelerated liver tumor formation as removal of GP73 alleviated the tumor burden in *HBV; Pten*^*−/−*^ mice.

Although prolonged HBV infection is the most frequent etiology in liver cancer, only a small fraction of HBV carriers eventually develop liver cancer after hepatic injury, inflammation, fibrosis and cirrhosis [9]. Additional molecular alterations are thus required for the malignant transformation of HBV infection. Various alterations of proto-oncogenes and tumor suppressors cause malfunction of PTEN-PI3K/AKT/mTOR signaling pathway in hepatic cancer [15, 16]. PTEN is the second most frequently altered tumor suppressor in human cancers [35]. PTEN expression is reduced in liver cancer which may be due to PTEN genetic mutations, transcriptional and post-transcriptional regulation alterations, and protein degradation [36]. Patients with reduced PTEN expression in liver cancer had poor prognosis [17]. Loss of *Pten* in mouse liver causes HCC around 18.5 months of age [21]. *Pten* inactivation causes ICC more frequently than HCC [37]. PTEN is a suppressor of AKT activity. AKT pathway alterations were observed in 96.5% of the tumors in *HBV* transgenic mice [31]. We and others identified that activation of β-catenin, AKT and c-jun are the important contributors of HCC development of *HBV* transgenic mice [31, 32, 38]. In this study, we found that liver-specific *Pten* deletion accelerated HBV-mediated liver tumor initiation and progression. In line with the reported findings [29, 30], *HBV* mice developed infrequent HCC around the age of 14 months. Half of *Pten*^*−/−*^ mice developed tumors by the age of 9 months. In contrast, liver tumors emerged at the age of 5 months and became malignant by the age of 8 months in *HBV; Pten*^*−/−*^ mice. These tumors exhibited pathologic characteristics of combined HCC-ICC. Compared to WT, *HBV* or *Pten*^*−/−*^ livers, *HBV; Pten*^*−/−*^ livers had enhanced apoptosis and severer liver injury. Increased phosphorylation of JNK was observed in *HBV; Pten*^*−/−*^ livers. Since JNK activation is a well-known apoptotic inducer in liver [39], it may partly contribute to the enhanced cell death in *HBV; Pten*^*−/−*^ livers. *HBV; Pten*^*−/−*^ mice not only illuminate the significance of *Pten* inactivation in HBV-associated liver tumor development but also represent a suitable model to investigate the pathologic and molecular mechanisms for mixed HCC-ICC.

Liver injury is usually followed by sustained inflammation and fibrosis. Fibrosis is a major pathological process of liver cancer [40]. IL-6 plays important role in chronic inflammation and has been demonstrated to promote diethylnitrosamine (DEN)-induced HCC development [41]. IL6 was increased in *HBV; Pten*^*−/−*^ livers. STAT3, a downstream target of IL6 [42], was also activated in *HBV; Pten*^*−/−*^ livers. Although no significant fibrosis was found in the livers of *HBV* or *Pten*^*−/−*^ mice, *HBV; Pten*^*−/−*^ livers had severe fibrosis. Thus, transgenic *HBV* and *Pten* deficiency synergistically promote hepatocyte death, liver injury, inflammation and fibrosis.

We present here that reduced PTEN and elevated GP73 associated with poor prognosis of HCC patients. GP73 expression is elevated by virus infection [22]. We and others have demonstrated that serum GP73 is a biomarker for liver cancer as its level increases during the progression of HBV infection, hepatic cirrhosis and tumor formation [23–25]. We reported that GP73 is an mTOR downstream effector and critical for xenografting HCC development and metastasis in immune-deficient nude mice [26]. GP73 also promotes xenografting HCC metastasis by regulating EGFR/RTK cell-surface recycling [27]. However, these studies were conducted on cellular experiments and xenografting tumors in nude mice. The role of GP73 in orthotropic liver cancer should be studied in immune-competent mice. We presented here that GP73 expression was enhanced in the livers and even higher in hepatic tumors of *HBV; Pten*^*−/−*^ mice. To study the involvement of GP73 in HBV-mediated orthotropic liver cancer development in immune-competent mice, we deleted GP73 in *HBV; Pten*^*−/−*^ mice. Compared with *HBV; Pten*^*−/−*^ mice, *HBV; Pten*^*−/−*^*; Gp73*^*−/−*^ mice had smaller and fewer tumor foci, indicating that GP73 is critical for the accelerated liver tumor formation in *HBV; Pten*^*−/−*^ mice. In accordance with the important role of GP73 in inflammation, knockout of GP73 reduced hepatocyte apoptosis and liver injury of *HBV; Pten*^*−/−*^ mice. Fibrosis was suppressed when GP73 was knocked out in *HBV; Pten*^*−/−*^ mice. Activated STAT3 and up-regulated IL6 were inhibited in *HBV; Pten*^*−/−*^*; Gp73*^*−/−*^ livers. These results explain why GP73 knockout blunted the synergistic effect of *HBV* transgene and *Pten* deficiency in promoting liver tumor initiation and progression. Therefore, potential inhibitors for GP73 expression and/or function such as GP73 antibodies may benefit liver cancer patients [26, 43].

## Conclusions

Our study provides the evidence of synergistic effect of *HBV* transgene and *Pten* deficiency in promoting mixed HCC-ICC initiation and progression in mice. Knockout of GP73 alleviates liver injury, inflammation and fibrosis, finally resulting in suppression of liver tumor development. *HBV; Pten*^*−/−*^ mouse is a good animal model for drug screening of liver cancer with PTEN dysregulation and HBV infection. Since GP73 is a promising target for liver cancer treatment, therapeutic regimens against GP73 should be developed and tested preclinically and clinically.

### Supplementary Information

**Additional file 1: Figure S1.** PCR genotyping of mouse tail genomic DNA.

## Data Availability

The datasets supporting the conclusions of this article are included within the article and its additional files.

## Abbreviations

HBV: Hepatitis B virus
HCC: Hepatocellular carcinoma
ICC: Intrahepatic cholangiocarcinoma
HCV: Hepatitis C virus
PTEN: Phosphatase and tensin homolog
PI3K: Phosphatidylinositol 3-kinase
GP73: Golgi membrane protein 73
GOLM1: Golgi membrane protein 1
H&E: Hematoxylin–eosin
ALT: Alanine aminotransferase
AST: Aspartate aminotransferase
DEN: Diethylnitrosamine
mTOR: Mechanistic target of rapamycin kinase
AKT: AKT serine/threonine kinase
TSC1/TSC2: TSC complex subunit 1/2
EGFR: Epidermal growth factor receptor
STAT3: Signal transducer and activator of transcription 3
IL6: Interleukin 6
