# Supplementary figures and images for: PTEN deficiency potentiates HBV-associated liver cancer development through augmented GP73/GOLM1
Source: J Transl Med. 2024 Mar 8;22:254. doi: 10.1186/s12967-024-04976-4 (PMC10924424; doi:10.1186/s12967-024-04976-4)

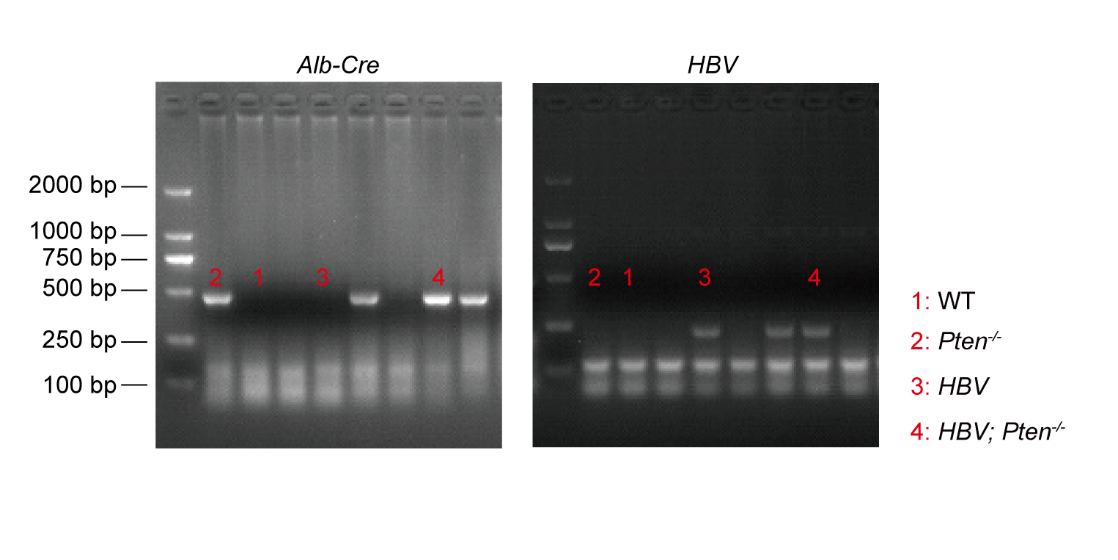


**Figure S1. PCR genotyping of mouse tail genomic DNA.**

Supplement: Supplementary file 1 — Additional file 1: Figure S1. PCR genotyping of mouse tail genomic DNA. [file 12967_2024_4976_MOESM1_ESM.docx]
